# Keep it simple: designing a user-centred digital information system to support chronic disease management in low/middle-income countries

**DOI:** 10.1136/bmjhci-2022-100641

**Published:** 2023-01-13

**Authors:** Daniel Burka, Reena Gupta, Andrew E Moran, Jennifer Cohn, Sohel R Choudhury, Tim Cheadle, Rahul Mullick, Thomas R Frieden

**Affiliations:** 1 Cardiovascular Health, Resolve to Save Lives, New York, New York, USA; 2 Department of Medicine, University of California San Francisco School of Medicine, San Francisco, California, USA; 3 Department of Medicine, Columbia University Irving Medical Center, New York, New York, USA; 4 Center for Global Health, University of Pennsylvania Perelman School of Medicine, Philadelphia, Pennsylvania, USA; 5 Department of Epidemiology and Research, National Heart Foundation Hospital and Research Institute, Dhaka, Bangladesh; 6 President, Resolve to Save Lives, New York, NY, USA

**Keywords:** Health Information Systems, Public health informatics, Medical Informatics Applications, Global Health

## Abstract

**Objective:**

Implement a user-centred digital health information system to facilitate rapidly and substantially increasing the number of patients treated for hypertension in low/middle-income countries.

**Methods:**

User-centred design of Simple, an offline-first app for mobile devices to record patient clinical visits and a web-based dashboard to monitor programme performance.

**Results:**

The Simple mobile application scaled rapidly over the past 4 years to reach more than 11 400 primary care facilities in four countries with over 3 million patients enrolled. Simple achieved median duration for new patient registration of 76 s (IQR 2 s) and follow-up visit entry of 13 s (IQR 1 s).

**Conclusions:**

A fast, easy-to-use digital information system for hypertension programmes that accommodates healthcare worker time constraints by minimising data entry and focusing on key performance indicators can successfully reach scale in low-resource settings.

## Introduction

Hypertension (HTN) (high blood pressure (BP)) is the world’s leading preventable cause of premature death, accounting for 10.7 million deaths per year, predominantly from heart attacks and strokes, with most deaths occurring in low/middle-income countries (LMICs).^1^ Large-scale HTN programmes in low-resource settings face challenges with overworked clinical staff and ineffective information systems.^2^ Since 2018, the WHO, the US Centers for Disease Control and Prevention, and Resolve to Save Lives have promoted the Global Hearts Initiative^3^ with five programme components: standard treatment protocols, consistent medication supply, team-based care, patient-centred care and information systems that facilitate real-time feedback on programme performance.^4^

HTN affects about one in four adults globally; substantial informatics capacity is needed to monitor and improve care for millions of patients. In many LMIC primary care facilities, staff spend hours daily recording and reporting data on inefficient paper-based systems. A well-designed digital HTN management system has immense potential to improve HTN care and save lives.

## Methods

The Simple mobile application, a free, open-source digital information system for large-scale HTN control programmes (www.simple.org), was launched in October 2018.^5^ Simple includes: (1) a mobile point-of-care app for healthcare workers to record patient visits and review treatment history (online supplemental figure 1); (2) a web-based dashboard for system managers to monitor programme performance across facilities and regions (online supplemental figure 2); and (3) tools that generate lists of patients overdue for care and send automated messages to promote continuity of care.

10.1136/bmjhci-2022-100641.supp1 Supplementary data

10.1136/bmjhci-2022-100641.supp2 Supplementary data

### User-centred design and implementation

The Simple app was developed following user-centred design principles.^6^ The initial prototypes were developed during a 5-day ‘Design Sprint’^7^ followed by three iterative rounds of testing at 30 facilities between November 2017 and July 2018 using the following methods: observing patient flows, task analysis with time-motion assessment and structured interviews with healthcare workers.^6^ We conducted interviews with 62 staff nurses, community health workers and medical officers using a structured interview guide, and observed users interacting and responding to high-fidelity prototypes. Interviews were recorded, transcribed and synthesised using thematic analysis.

Table 1 summarises key design considerations compiled from healthcare worker interviews and our direct observations. When asked what they want in a digital tool, healthcare workers’ primary recommendation was to ensure rapid data recording so they could focus on clinical care. We observed that clinicians have 3–4 min per patient, so we committed to a goal of under 20 s—10% of a typical clinical visit—for data entry. This informed adoption of a limited set of data elements (online supplemental table 1). We designed for mobile devices due to inconsistent internet observed and user preference. Healthcare workers, who often painstakingly compile lists of overdue patients from paper records, requested automated overdue lists to contact patients.

10.1136/bmjhci-2022-100641.supp5 Supplementary data

**Table 1:** Key user-centred considerations for mobile application design for hypertension management in low/middle-income country programmes

Minimise data entry	Set targets for speed and usability, for example, less than 80 s for new patient registration and initial clinical visit and less than 20 s for follow-up patient visits Resist temptation to add features or data requirements unless speed and ease-of-entry targets can be maintained
Offline-first	Faster and more reliable, with less chance of patients not being registered or follow-up information not updated because of slow or absent connectivity Minimise data elements to be stored and avoid large data components, for example, photos or scans, to ensure capacity for storing data offline on mobile devices
Use QR codes for patient identification	This low-tech approach can cut more than 20 s off patient look-up time and reduce errors
Maximise usability	Design screens and interfaces based on actual user testing, not what programme experts or engineers think staff will want Use straightforward information design to provide providers and programme managers with visualisation of the most important indicators of programme progress The design and accessibility for Simple follow best-in-class standards, such as the US Digital.gov guidelines (https://digital.gov/resources) Prioritise usability for front-line staff over interoperability across different platforms
Easy to train in clinic	Training in 1 hour in clinics supports rapid scale-up and allows for healthcare workers to train each other, given high staff turnover in public facilities
Data governance and security	A local or national government agency (eg, Ministry of Health) owns the data The system must have clear and ethical data governance from an early stage and address ownership transparently, such as through Memorandum of Understanding agreements outlining governance with each country’s Ministry of Health Mobile device data are encrypted both in transit and at rest using industry-standard security best practices Patients should have control of their own data, allowing them to monitor and improve their health, and must opt in at enrolment to consent to have their data collected
Enable mobile device use	A device that is always with providers is more likely to be charged, have up-to-date software, connect with the internet at least periodically and be used by healthcare workers
Enable patient registers for recall to care	This can improve patient adherence to treatment by facilitating tracking of progress and patient outreach to the entire patient panel by programme staff
Build for scale	Build to maintain speed and performance with successful scale-up to handle large numbers of patients

Healthcare workers also informed features that were ultimately abandoned (ie, patient lookup by age, patient risk scoring and facility ranking). We minimised error checking and pop-up reminders based on user testing with healthcare workers frustrated by these interruptions. We enabled an in-app ‘progress’ view of key indicators and sent monthly dashboard reports to key stakeholders via email and text message, as healthcare workers and managers did not reliably access online dashboards.

The team conducts monthly user interviews to continuously inform the iterative development process. To date, 62 user studies with approximately 240 interviews have been conducted to test new features pre-development, usability test completed features and explore future designs.

### Technical architecture

The data collection tool is a native Android application, which relies heavily on local SQLite database usage (ie, an ‘offline-first’ approach). This strategy ensures reliable, fast performance despite unreliable internet connections. The Simple dashboard is a web-based tool with responsive design, enabling data use from computers or mobile devices. Modern RESTful APIs communicate data between the server and devices. The app is designed to be lightweight (31.29 MB); even with patient data from a local region synced to local storage for offline access, the app is still relatively small (~100 MB with 36 000 patients).

Simple was evaluated in three areas: (1) scale (uptake measured by numbers of active implementing facilities and patients enrolled); (2) speed (time to record new patient registrations and follow-up visits from app metrics metadata; health worker time differences between facilities using paper records vs Simple); and (3) outcomes (BP control: proportion of patients with BP <140/90 in the previous 3 months, before and after implementation).

## Results

### Scale

From October 2018 to November 2022, Simple was implemented in more than 11 400 public hospitals and clinics in four countries (India, Bangladesh, Ethiopia and Sri Lanka) to manage more than 3 million patients (online supplemental figure 3).^8^

10.1136/bmjhci-2022-100641.supp3 Supplementary data

### Speed

Simple achieved a median time to register new patients of 76 s (IQR 2 s) and enter follow-up visits in 13 s (IQR 1 s) (online supplemental table 2). A time-motion study demonstrated that healthcare workers at facilities using Simple averaged 24 min less each workday to enter and manage clinical data compared with facilities using paper records (15 min vs 39 min, p<0.001).^9^

10.1136/bmjhci-2022-100641.supp6 Supplementary data

### Outcomes

A pre/post-comparison of BP control rates before and after implementation of the Simple app in Bangladesh demonstrated that BP control increased from 20% (1567 patients with controlled BP of 7787 patients enrolled as of February 2020) before Simple to 39% (4555 patients with controlled BP of 11 728 patients enrolled as of August 2020) 6 months after Simple implementation (online supplemental figure 4).

10.1136/bmjhci-2022-100641.supp4 Supplementary data

A quality improvement (QI) strategy based on Simple dashboard data likely contributed to increased BP control. For example, the dashboard identified a high 3-month loss to follow-up rate (50%) as a primary driver of low BP control. The main QI intervention in the 6 months after Simple implementation in Bangladesh was contacting patients overdue for care using the app’s overdue list and secure calling features, with the 3-month loss to follow-up rate decreasing from 50% to 26% after 6 months.

## Discussion

Experience with successful uptake of the Simple mobile application demonstrates that fast, easy-to-use clinical software can scale widely to support large-scale HTN control programmes in LMICs. Most digital tools in these settings are time-consuming and rarely prioritise user experience. We applied user-centred design with rigour to create a practical digital tool. We listened to healthcare workers whose primary concern was that using the app would make their jobs harder or longer. Four key principles emerged from our observations and interviews with healthcare workers.

### Principle 1: very fast and easy to use

A digital health information system for chronic disease control programmes must be fast and easy to use by healthcare staff as LMIC patient encounters are often under 5 min. Training also takes less time when the application is uncomplicated and intuitive. Training users in situ in hospitals and clinics in under an hour reduces overhead training costs, facilitates train-the-trainer models in areas of frequent staff turnover and accelerates scale.

### Principle 2: minimal data entry

A limited set of data elements can be entered quickly yet provide essential insights to improve care. Including extraneous data elements results in software that is never adopted, is used inconsistently or collects inaccurate data. Healthcare workers spend nearly half the time per day on data entry at facilities using Simple compared with facilities using paper records.

### Principle 3: offline-first

Data entry software that is offline-first (offline entry with data synchronised when internet access is sufficient) is needed where connectivity is intermittent but also increases efficiency for places with wi-fi. Internet-enabled applications that are not offline-first must constantly connect with central servers to transmit data, which can result in sluggish performance.^10^

### Principle 4: limit dashboards to key indicators that drive QI

Simple prioritises three core indicators used to identify gaps in performance and drive continuous QI: patients in care with controlled BP; patients who missed scheduled visits and patients enrolled as a percentage of the total estimated hypertensive population (estimated programme coverage). Providing programme managers with monthly data on these three indicators supports identification of struggling facilities and implementation of programme improvements. The primary goal of a digital health information system for a chronic disease programme is to function as a support for clinicians and programme managers by providing feedback loops to improve quality.^11^

### Limitations

There are limitations to the Simple mobile application and to this assessment. The Simple app does not contain comprehensive health information on each patient. It is available only for Android devices. We have not deployed Simple to private sector healthcare providers. Simple includes a diabetes module, which is not described here. Our pre/post-comparison of BP control in Bangladesh is not a rigorous assessment of the independent contribution of Simple to improved HTN control and other patient outcomes; a formal evaluation is currently underway.

## Conclusion

A digital system to manage HTN control programmes that accommodates healthcare worker time constraints by minimising data entry and focusing on key performance indicators can reach scale in low-resource settings. The system must be designed with a focus on simplicity, speed and scale with front-line staff usability prioritised from the start. The best system is ultimately one that helps the most patients control their BP.

## Data Availability

All data relevant to the study are included in the article or uploaded as supplemental information. All referenced data are derived from published material and can be accessed where cited.
