# Supplementary figures and images for: Keep it simple: designing a user-centred digital information system to support chronic disease management in low/middle-income countries
Source: BMJ Health Care Inform. 2023 Jan 13;30(1):e100641. doi: 10.1136/bmjhci-2022-100641 (PMC9843217; doi:10.1136/bmjhci-2022-100641)

Supplemental Figure 1. Simple mobile application workflow at the point of care

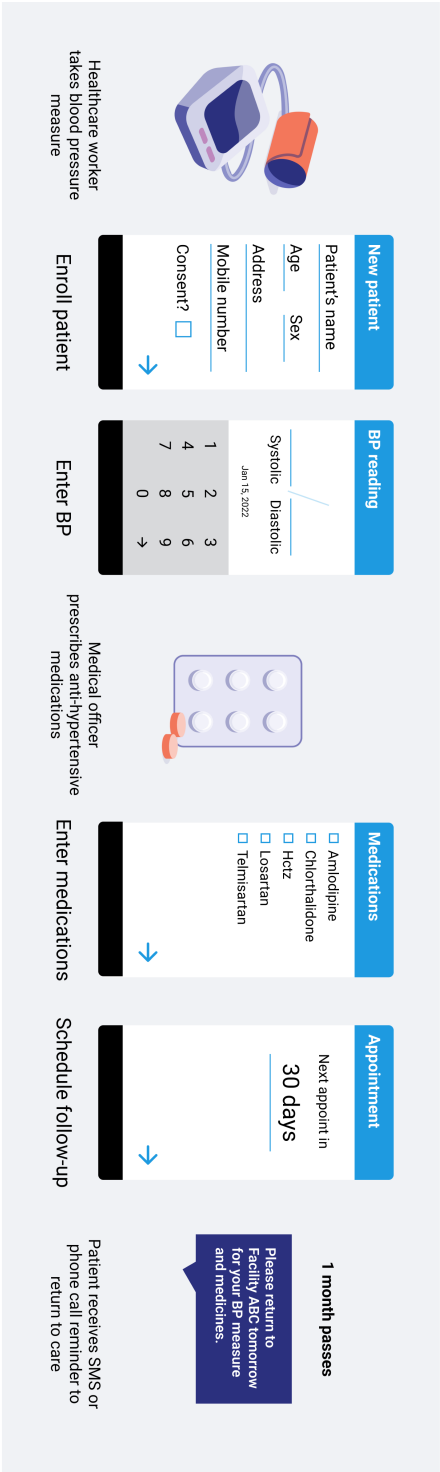

Supplement: Supplementary data [file bmjhci-2022-100641supp001.pdf]

**Supplemental Figure 3. Scale-up implementation timeline of the Simple mobile application**

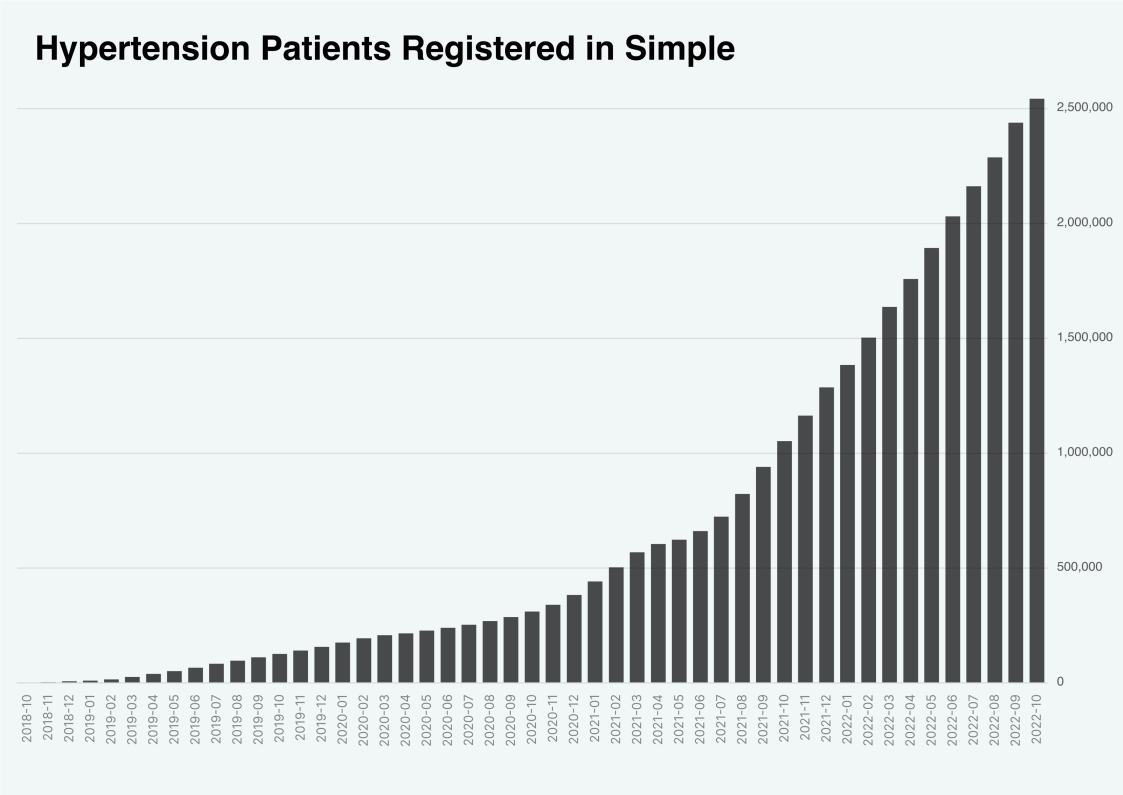

Supplement: Supplementary data [file bmjhci-2022-100641supp003.pdf]
