# Supplementary material for: Keep it simple: designing a user-centred digital information system to support chronic disease management in low/middle-income countries
Source: BMJ Health Care Inform. 2023 Jan 13;30(1):e100641. doi: 10.1136/bmjhci-2022-100641 (PMC9843217; doi:10.1136/bmjhci-2022-100641)
Supplement: Supplementary data [file bmjhci-2022-100641supp002.pdf]

Supplemental Figure 2. Simple mobile application data dashboard

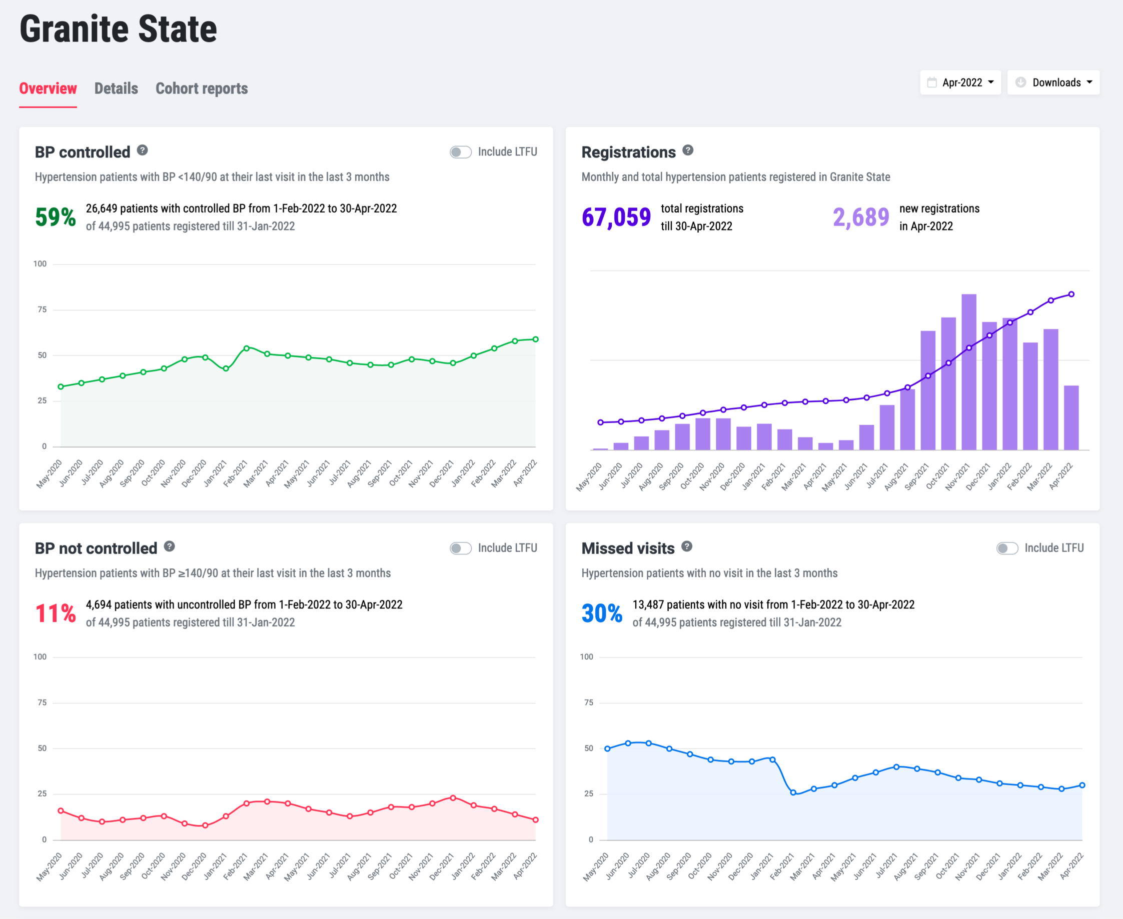

Note: Data shown are for illustrative purposes to demonstrate display of key indicator trends and do not represent actual program data.
