# Supplementary material for: Keep it simple: designing a user-centred digital information system to support chronic disease management in low/middle-income countries
Source: BMJ Health Care Inform. 2023 Jan 13;30(1):e100641. doi: 10.1136/bmjhci-2022-100641 (PMC9843217; doi:10.1136/bmjhci-2022-100641)
Supplement: Supplementary data [file bmjhci-2022-100641supp005.pdf]

Supplemental Table 1. Minimal data elements used by the Simple mobile application

Data Type	Detail
**Basic patient profile**	Name, sex, age, address, telephone number
**Unique ID**	Where possible, use a local health ID; a UUID-based QR code identifier can be used in places without a usable ID system
**Key comorbidities**	Basic "yes/no" questions facilitate review of cardiovascular disease history (i.e.., diabetes, heart attack, stroke, kidney disease)
**Blood pressures**	Record all systolic and diastolic measurements in standardized format, with error checking for physiologically impossible values
**Current medications**	Many strong hypertension control programs choose simplified treatment protocols;[*,†] a digital health system can track each patient's current medications, facilitate clinical decision support, and review adherence to protocols
**Follow-up appointment**	Recording a patient's next expected clinical appointment enables sending reminders and improving patient retention in care; recording how many patients were contacted is a good measure of health worker responsiveness and can be used for quality improvement
**Drug stock**	Monthly data on stock received and end-of-month stock on hand in the facility pharmacy, if applicable; inconsistent drug supply and stockouts are major barriers to chronic disease treatment

* Frieden TR, Varghese CV, Kishore SP, et al. Scaling up effective treatment of hypertension – A pathfinder for universal health coverage. *J Clin Hypertens (Greenwich)* 2019;21:1442-1449. doi: 10.1111/jch.13655.

† Jaffe MG, Frieden TR, Campbell NRC, et al. Recommended treatment protocols to improve management of hypertension globally: A statement by Resolve to Save Lives and the World Hypertension League (WHL). *J Clin Hypertens (Greenwich)* 2018;20:829-836. doi: 10.1111/jch.13280.
