# Supplementary material for: Keep it simple: designing a user-centred digital information system to support chronic disease management in low/middle-income countries
Source: BMJ Health Care Inform. 2023 Jan 13;30(1):e100641. doi: 10.1136/bmjhci-2022-100641 (PMC9843217; doi:10.1136/bmjhci-2022-100641)
Supplement: Supplementary data [file bmjhci-2022-100641supp006.pdf]

Supplemental Table 2. Healthcare worker documentation time for recording new patient and follow-up patient visits in the Simple app

	New patient registration Median (IQR)	Follow-up patient visit Median (IQR)
**Scan ID card**	6 seconds (0 seconds)	2 seconds (0 seconds)
**Enter patient demographics, cardiovascular history, current medications**	59 seconds (1 second)	---
**Enter blood pressure measurement**	5 seconds (0 seconds)	5 seconds (0 seconds)
**Update Medications**	5 seconds (1 second)	5 seconds (1 second)
**Schedule follow-up visit**	1 seconds (0 seconds)	1 seconds (0 seconds)
**Total**	76 seconds (2 seconds)	13 seconds (1 second)
