# Supplementary material for: Keep it simple: designing a user-centred digital information system to support chronic disease management in low/middle-income countries
Source: BMJ Health Care Inform. 2023 Jan 13;30(1):e100641. doi: 10.1136/bmjhci-2022-100641 (PMC9843217; doi:10.1136/bmjhci-2022-100641)
Supplement: Supplementary data [file bmjhci-2022-100641supp004.pdf]

Supplemental Figure 4. Blood pressure control rates in Bangladesh, before and after adoption of Simple mobile application

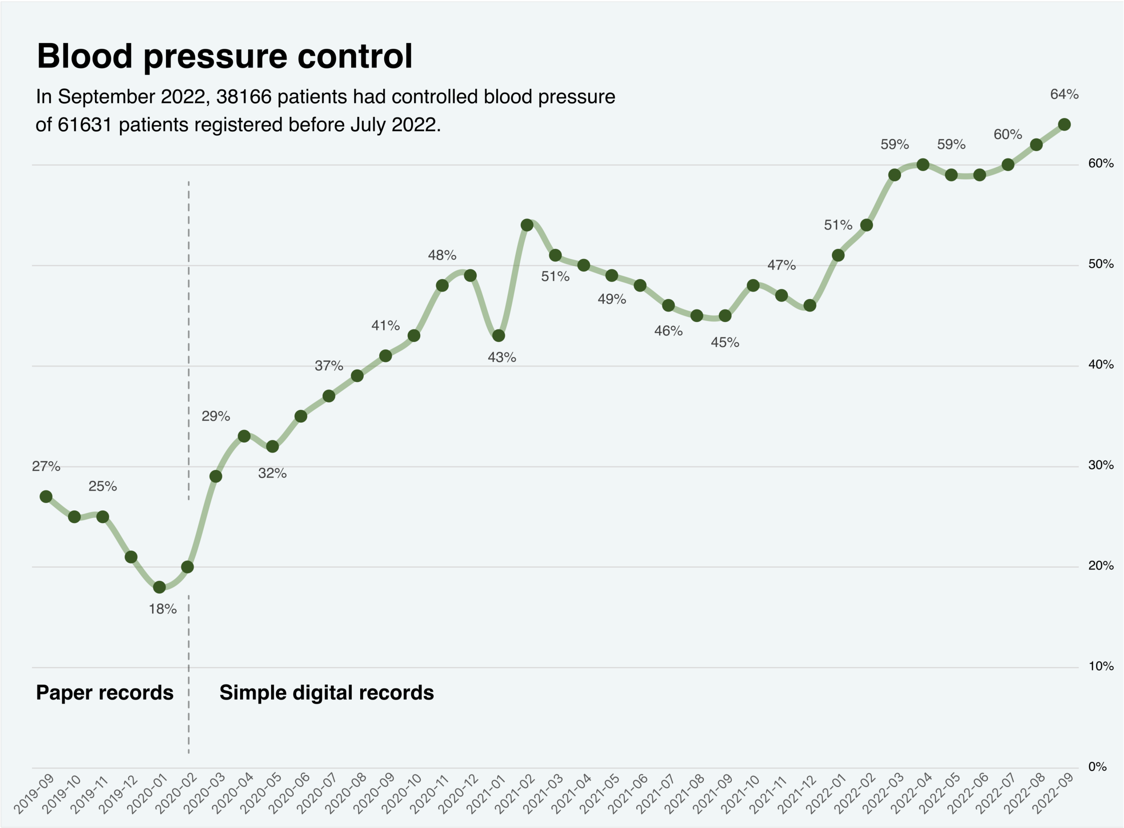

Source: National Heart Foundation of Bangladesh (unpublished data).

Note: Data are for four health centers in Sylhet, Bangladesh that implemented the Simple mobile application in February 2020. BP control increased from 20% (1,567 patients with controlled BP of 7,787 patients enrolled as of February 2020) before Simple to 39% (4,555 patients with controlled BP of 11,728 patients enrolled as of August 2020) six months after implementation.
